# Do the P1 and P2 hairpins of the Guanidine-II riboswitch interact?

**DOI:** 10.1093/nar/gkaa703

**Published:** 2020-08-28

**Authors:** Christine Wuebben, Maria F Vicino, Marcel Mueller, Olav Schiemann

**Affiliations:** Institute of Physical and Theoretical Chemistry, University of Bonn, Wegelerstr. 12, 53115 Bonn, Germany; Institute of Physical and Theoretical Chemistry, University of Bonn, Wegelerstr. 12, 53115 Bonn, Germany; Institute of Physical and Theoretical Chemistry, University of Bonn, Wegelerstr. 12, 53115 Bonn, Germany; Institute of Physical and Theoretical Chemistry, University of Bonn, Wegelerstr. 12, 53115 Bonn, Germany

## Abstract

Riboswitches regulate genes by adopting different structures in responds to metabolite binding. The guanidine-II riboswitch is the smallest representative of the ykkC class with the mechanism of its function being centred on the idea that its two stem loops **P1** and **P2** form a kissing hairpin interaction upon binding of guanidinium (Gdm^+^). This mechanism is based on in-line probing experiments with the full-length riboswitch and crystal structures of the truncated stem loops **P1** and **P2**. However, the crystal structures reveal only the formation of the homodimers **P1 | P1** and **P2 | P2** but not of the proposed heterodimer **P1 | P2**. Here, site-directed spin labeling (SDSL) in combination with Pulsed Electron–Electron Double Resonance (PELDOR or DEER) is used to study their structures in solution and how they change upon binding of Gdm^+^. It is found that both hairpins adopt different structures in solution and that binding of Gdm^+^ does indeed lead to the formation of the heterodimer but alongside the homodimers in a statistical 1:2:1 fashion. These results do thus support the proposed switching mechanism.

## INTRODUCTION

Riboswitches are RNA elements that regulate gene expression and are found in the 5′-untranslated regions of mRNAs. These highly structured RNA sequences are characterized by two components, the aptamer domain and the expression platform. The aptamer domain is able to bind metabolites in a selective manner through its conserved sequence. Binding of these molecules leads to structural changes in the expression platform and thereby to an altered expression level of the downstream gene. A variety of mechanisms for such a gene regulation are known, with the most common ones being transcription termination and translation initiation (1–3). Normally, the metabolite corresponding to the riboswitch is predicted based on the gene set regulated by the riboswitch. However, there are candidate riboswitches for which ligand determination is still challenging (4). One of the orphan riboswitches was the *ykkC* element first reported by Barrick *et al.* (5). Later, computational studies identified, beside the transcriptionally acting *ykkC*, structurally distinct motifs regulating a similar set of genes but in a translational manner, the *mini-ykkC* and the *ykkC*-III (6,7). In 2017, the Breaker laboratory determined guanidinium (Gdm^+^) as the ligand and the riboswitch family was renamed after this ligand, yielding the guanidine-I riboswitch (*ykkc*), the guanidine-II riboswitch (*mini-ykkC*) and the guanidine-III riboswitch (*ykkC-III*) (8,9).

**Figure 1.**
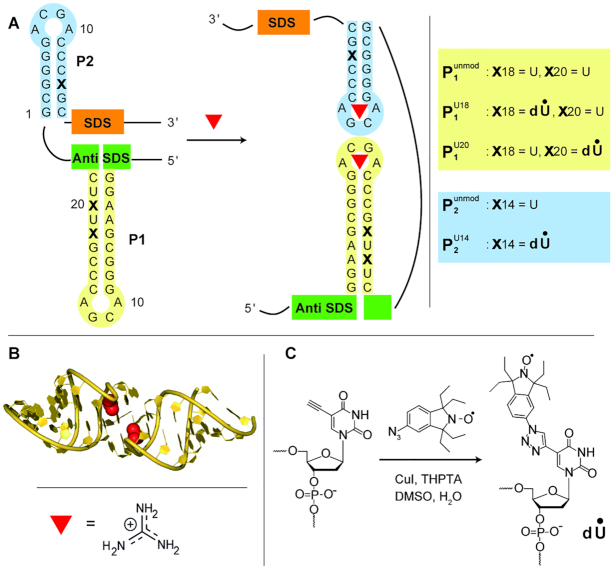
Switching mechanism, structure elements and labeling of the guanidine-II riboswitch. (**A**) Proposed switching mechanism of the guanidine-II riboswitch (11). ‘SDS’ denotes the Shine-Dalgarno Sequence and ‘Anti-SDS’ the complementary Anti-Shine-Dalgarno Sequence. The hairpins **P1** and **P2** are color coded in yellow and blue, respectively. The modified positions are marked with an X. The modifications and the corresponding abbreviations of the RNA sequences are shown next to it. The subscripts 1 or 2 denote stem loop **P1** or **P2**, respectively. The superscript denotes the base that is spin labeled. If it reads ‘unmod’, the strand is not labeled. (**B**) Crystal structure of the homodimer **P1** (pdb: 5NDI, 10) and the chemical structure of the guanidinium cation (Gdm^+^). (**C**) Scheme of the spin labeling reaction performed.

The guanidine-II riboswitch is the smallest representative of the ykkC family and consists of the two stem loops **P1** and **P2** (marked in Figure 1A), which are connected via a flexible linker of variable length (7–40 nucleotides) (6,9). The full-length riboswitch has not been crystalized yet, but crystal structures of the truncated **P1** (*Escherichia coli* and *Gloeobacter violaceus*, Figure 1B) (10) and **P2** hairpin elements (*Pseudomonas aeruginosa*) (11) revealed, that they form both kissing-loop homodimers upon binding of Gdm^+^, with each tetraloop binding one Gdm^+^. In combination with in-line probing experiments (12) and fluorescence measurements (13) hinting at a **P1 $|$ P2** interaction, a switching model was proposed in which the **P1** and **P2** stem loops form an intra-strand **P1 $|$ P2** kissing loop interaction (11), which leads to translational promotion of genes modifying or transporting Gdm^+^ (Figure 1A). However, the mechanistically important **P1 $|$ P2** heterodimer formation has not been proven, yet.

Pulsed Electron-Electron Double Resonance (PELDOR or DEER) (14–17) is a pulsed Electron Paramagnetic Resonance (EPR) (18) method for measuring distances between two unpaired electrons in frozen solution. Commonly, stable nitroxide radicals are used as the electron bearing centers, which can be site-selectively attached to e.g. RNAs via various different methods called site-directed spin labeling (SDSL) (19). The advantages of PELDOR are: it has no restriction with respect to the size of the biomolecule (20), it provides distances in the range of 1.5–16 nm (21-23) in form of distance distributions that mirror the conformational flexibility/distribution of the biomolecule in liquid solution (23), and it has a precision of 0.1 nm depending on the data quality (24). Thus, if the structure or dynamics of a biomolecule changes e.g. upon binding of a ligand, the distance distribution will change accordingly. Over the years several ribozymes and riboswitches have been studied with respect to their structures and conformational changes by SDSL/PELDOR (25–29). Here, the SDSL/PELDOR approach is used to probe for the proposed Gdm^+^ induced **P1 $|$ P2** heterodimer formation between the truncated hairpins **P1** and **P2** and thereby to test the mechanism of the switching model.

## MATERIALS AND METHODS

### RNA preparation

The RNA constructs were purchased from metabion international AG (Planegg, Germany) with the denoted sequences in Supplementary Table S1. Concentration determination was done by absorption measurement and calculated with the extinction coefficients provided by the provider (Supplementary Table S2). The RNA spin labeling reaction was carried out according to a previous published method (30). 8 μl of a 250 mM solution of tris(3-hydroxypropyltriazolylmethyl) amine (THPTA, Sigma–Aldrich) in dimethylsulfoxide (DMSO, Carl Roth) were mixed with 8 μl of a freshly prepared 50 mM solution of copper-I-iodide (Carl Roth) in DMSO. Then, 20 μl of DMSO were added and the mixture was incubated for 5 min at room temperature. Meanwhile, 2.5 nmol of the dried oligonucleotide with a 5-ethynyl-2′-deoxy-uridine at the desired position in the sequence was resuspended in 4.4 μl diethylpyrocarbonate (DEPC, Carl Roth) treated water. Then, 3.6 μl of the catalytic solution and 2 μl of a 100 mM spin label solution in DMSO were added to the RNA solution. The reaction mixture was mixed and incubated for 30 min at 60°C and 300 rpm (Thermomixer comfort, Eppendorf, Hamburg, Germany). The reaction was quenched by adding 480 μl Milli-Q water and desalted via an Amicon© ultra 3K column (Merck). The desalted products were finally purified via reverse-phase high-performance liquid chromatography with an Agilent 1200 Series HPLC System (Agilent Technology, Santa Clara, CA, USA) in combination with the Zorbax 300SB-C18, 4.6 × 150 mm column (Agilent Technologies, Santa Clara, CA, USA). The RNA constructs were purified at 60°C column temperature. As the eluent 0.1 M triethylammoniumacetate was used with a gradient of 8% → 25% acetonitrile in 20 min (Supplementary Figure S1). The desired fractions were desalted with an Amicon© ultra 3K column (Merck). The final yields are listed in Supplementary Table S3 and vary between 60 and 72% depending on the RNA construct.

### LCMS

LCMS measurements were performed on an *HTC esquire* from Bruker Daltonik in combination with an Agilent 1100 Series HPLC system (Agilent Technologies) using a Zorbax Narrow Bore SB C18 (2.1 × 50 mm, 5 μm) column (Agilent Technologies). 10 mM triethylamine/100 mM hexafluoro-isopropanol was used as the solvent with a gradient of 5% → 20% acetonitrile in 20 min (Supplementary Figure S2 and Supplementary Table S4).

### cw EPR spectroscopy

For the EPR sample preparation, the RNA was resuspended into folding buffer (10 mM HEPES, 10 mM KCl, 10 mM MgCl_2_; pH 7.5) in the absence or presence of 40 mM Gdm^+^. The samples were incubated at 95°C for 5 min and subsequently cooled on ice for 10 min. In the following, the Gdm^+^ concentrations are abbreviated as ?–Gdm^+^ (no Gdm^+^), +Gdm^+^ (0.4 mM Gdm^+^) and ++Gdm^+^ (40 mM Gdm^+^). cw X-Band EPR spectra were recorded on an EMXmicro spectrometer from Bruker BioSpin (Rheinstetten, Germany). The spectra were measured with a microwave power of 2 mW, a modulation frequency of 100 kHz, a modulation amplitude of 1 G, a microwave frequency of 9.6 GHz, and 1300 points in the field interval of 337 - 350 mT (Supplementary Figure S3 and Supplementary Table S5).

### Pulsed EPR and PELDOR

The pulsed Q-band EPR measurements were conducted on 25 μM samples in deuterated buffer containing 20% ethylene glycol-d_6_. The spectrometer was an ELEXSYS E580 EPR spectrometer (Bruker BioSpin, Rheinstetten, Germany) equipped with an ER 5106QT-II resonator and a 150 W TWT-amplifier (Applied System Engineering, Fort Worth, TX, USA). The temperature was adjusted to 50 K using a CF935 helium gas-flow cryostat (Oxford Instruments, Abingdon, UK) in conjugation with an Oxford Instruments ITC 502 temperature controller. The PELDOR experiments were performed with the standard four-pulse sequence. The frequency of the pumping pulse was set at the maximum intensity of the nitroxide signal. The offset between pump and detection frequencies was varied as depicted in Supplementary Figure S4 and denoted in Supplementary Table S6. π/2- and π-pulse lengths of 12 and 24 ns were used. For the π/2-pulse, a two-step phase cycle was executed. The pump pulse length was set to the optimal length. The initial τ was set to 260 ns. Deuterium modulation was suppressed by addition of eight time-traces with an increment of 16 ns for τ. The detection window with a width of 40 ns was set to the maximum of the echo. To achieve an acceptable SNR, the signal was averaged for 3 to 24 h. The shot repetition time was set to 3 ms. All original time traces are shown in Supplementary Figure S5. DeerAnalysis2016 (31) was used to process the summed data. The zero time was set to the maximum of the time trace before fitting the homogenous background decay function. Tikhonov Regularization was used to extract the probability function for the distance distribution using the L-curve as a criterion for determining the optimum regularization parameter (Supplementary Figures S6 and S7). The validated PELDOR data are shown in Supplementary Figure S8.

### *In silico* spin labeling with MtsslWizard

The *in silico* predicted distances were generated by attaching the nitroxide spin label to the uridine base in the respective crystal structure within mtsslWizard, which then generates the nitroxide rotamers and extracts the label-to-label distance measured from N-to-N (32–34). For **P2**, the crystal structure of pdb: 5VJ9 was used, whose sequence is derived from *P. aeruginosa* (11). For **P1**, the crystal structure of pdb: 5NDI was used, whose sequence is derived from *E. coli* (10). Therefore, a uridine was mutated into the labeling position with 3DNA (35). In order to determine the distance of the heterodimers, the desired bases were also mutated within 3DNA. A-Form RNA duplexes were also generated with 3DNA (35).

### CD spectroscopy

CD-spectra were recorded on a JASCO J-810 spectropolarimeter at room temperature with an 0.01 cm cell by averaging over 15 scans. The scanning speed was adjusted to 200 nm/min and the spectral range to 200–320 nm. The measurements were performed on 10 μM samples in the case of **P1** and 25 μM samples of **P2**. The samples were annealed in 10 mM HEPES, 10 mM MgCl_2_, 10 mM KCl (pH 7.5) in the presence of 40 mM Gdm^+^ to 95°C for 5 min with subsequently cooling on ice for 10 min (Supplementary Figure S9).

### *T*_m_ measurements

UV–VIS based melting curves were recorded on a Cary 100 UV–VIS spectrophotometer (Agilent Technologies) at a wavelength of 260 nm. The temperature of the sample was increased with a heating rate of 1°C/min from 20°C to 95°C. The measurements were performed on 4 μM samples in the case of **P1** and 25 μM samples in the case of **P2**, due to its lower absorbance. The samples were annealed in 10 mM HEPES, 10 mM MgCl_2_, 10 mM KCl (pH 7.5) in the presence of 40 mM Gdm^+^ at a temperature of 95°C for 5 min and subsequent cooling on ice for 10 min (Supplementary Figure S10 and Supplementary Table S7).

### Native PAGE

Native PAGE (15%) analysis was carried out on 90 pmol of RNA. The samples were annealed in 10 mM HEPES, 10 mM MgCl_2_, 10 mM KCl (pH 7.5) in the absence or presence of 40 mM Gdm^+^ by heating to 95°C for 5 min followed by cooling on ice for at least 10 min. Finally, glycerol was added in equal ratio to the sample volume. Tris-borate buffer (89 mM Tris base, 89 mM boric acide, 25 mM NaCl) in the absence (Supplementary Figure S11) or presence of 40 mM Gdm^+^ (Supplementary Figure S12) was employed as running buffer. Staining was performed with GelRed (1:10000, Biotium, Hayward, CA, USA).

## RESULTS

### Characterization

**Figure 2.**
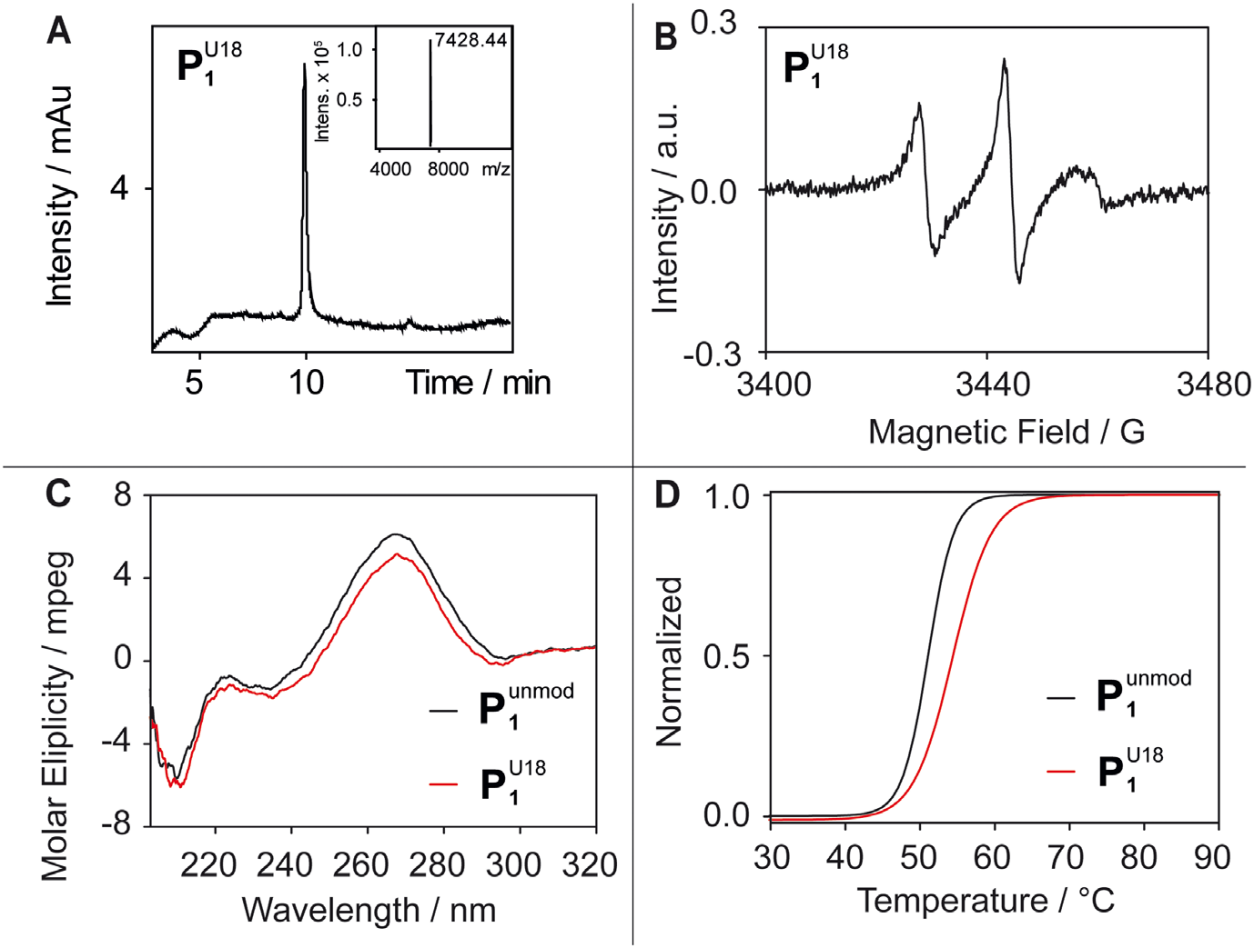
Characterization of ${\rm{P}}_{\rm{1}}^{{\rm{U18}}}$. (**A**) LCMS Analysis of the spin labeled and purified RNA constructs. The UV-trace was recorded at 260 nm and the inset shows the deconvoluted ESI^−^-mass data. The calculated mass *M*_calc_(${\rm{P}}_{\rm{1}}^{{\rm{U18}}}$) = 7365.23 matches the found mass *M*_found_(${\rm{P}}_{\rm{1}}^{{\rm{U18}}}$) = 7428.44, which is assigned to [M+^23^Na+^39^K]^+^. (**B**) cwEPR spectra of ${\rm{P}}_{\rm{1}}^{{\rm{U18}}}$. (**C**) CD spectra and (**D**) UV-VIS melting curve of the unmodified ${\rm{P}}_{\rm{1}}^{{\rm{unmod}}}$ (black line) and labeled ${\rm{P}}_{\rm{1}}^{{\rm{U18}}}$ (red line) in the presence of Gdm^+^. *T*_m_(${\rm{P}}_{\rm{1}}^{{\rm{unmod}}}$) = 51.0°C and *T*_m_(${\rm{P}}_{\rm{1}}^{{\rm{U18}}}$) = 54.4°C.

Several strategies have been developed for the site-specific spin labeling of RNA strands at the sugar (36–39), the phosphate (40–43) or the base moiety (30,44–51). The bioconjugation can either be performed during the oligonucleotide synthesis or through post synthetic spin labeling of pre-functionalized sites in the oligonucleotide. Here, a previously published protocol (30) is utilized in which a 5-ethynyl-2′-deoxy-uridine is incorporated into the RNA sequence during automated phosphoramidite synthesis. The prefunctionalized strands are then reacted in solution with an azide functionalized 1,1,3,3-tetraethylisoindoline spin label by way of a copper(I)-catalyzed azide–alkyne cycloaddition (CuAAC) (Figure 1C). This ‘click’-chemistry based method was chosen because it has a short reaction time, can be performed in solution, has a convenient purification protocol and provides high yields (28,30,48). Using this procedure, the RNA constructs in Figure 1A were synthesized, whose base sequences are derived from the *Pseudomonas aeruginosa* guanidine-II riboswitch. All of the spin labeled RNAs carry a single spin label only and were tested for labeling degree, purity and the influence of the label on the structure. Exemplary, this is shown for ${\rm{P}}_{\rm{1}}^{{\rm{U18}}}$ in Figure 2, the other data are collected in the SI. The successful incorporation of the spin label in ${\rm{P}}_{\rm{1}}^{{\rm{U18}}}$ as well as the purity of the sample was confirmed by liquid chromatography–mass spectrometry (LCMS) showing only one peak in the chromatogram and only one peak in the ESI-mass, which corresponds to the calculated mass (Figure 2A). The yield of purified ${\rm{P}}_{\rm{1}}^{{\rm{U18}}}$ was 60% with respect to the starting amount of RNA and varies in general between 60 and 72% depending on the RNA sequence (see Supplementary Table S4). Continuous wave (cw) EPR measurements were used to confirm quantitative labeling via spin counting and to verify the absence of free label (Figure 2B). The influence of the spin label on the RNA structure was assessed via CD-spectroscopy, UV–VIS based RNA melting studies and native PAGEs. The CD spectra of unmodified and labeled **P1** show very similar negative and positive molar ellipticity at 210 and 270 nm, and the melting temperature increased by 3.4°C (Figure 2C, D), which is in the same range as found in the literature before for this and other spin labels (28,30,46). The band shifts in the native PAGEs are in all cases the same for the unmodified and labeled RNA (Supplementary Figures S11 and S12). It was further tested whether conformational changes can be detected for **P1** and **P2** upon adding Gdm^+^ and indeed a band shift can be observed that is the same for unmodified and labeled RNA. Based on these data, the degree of perturbation by the label is judged as being only minor and local.

### PELDOR measurements

**Figure 3.**
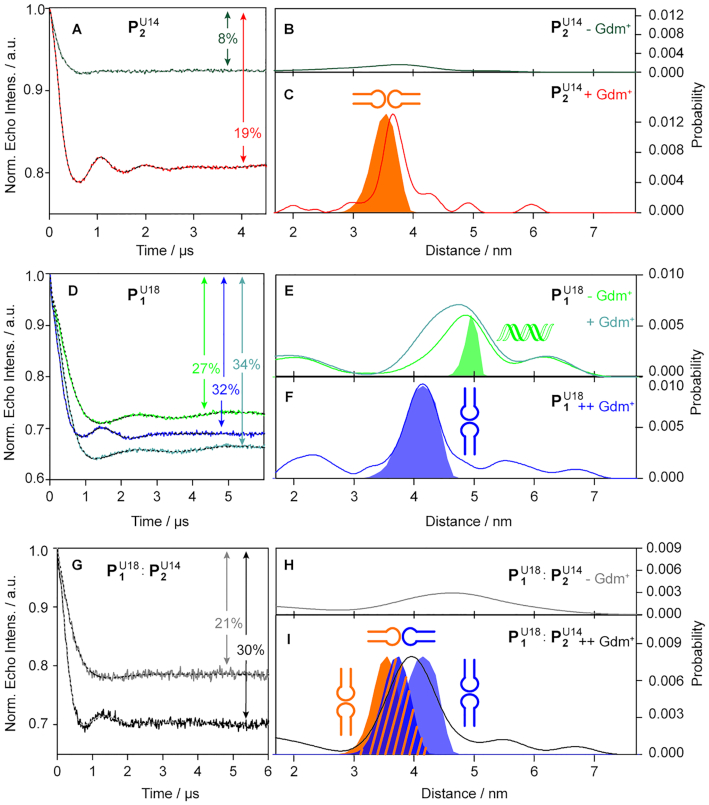
PELDOR time traces, distance distributions and assigned structures for ${\rm{P}}_{\rm{2}}^{{\rm{U14}}}$ and ${\rm{P}}_{\rm{1}}^{{\rm{U18}}}$. Top panel: (**A**) Background corrected PELDOR time traces for ${\rm{P}}_{\rm{2}}^{{\rm{U14}}}$ in the absence (dark green line) and presence (orange line) of Gdm^+^. The fits are overlaid as grey or black dotted line, respectively. The modulation depths are indicated in percentages. (**B** and **C**) The distance distributions corresponding to the time traces in A) shown as solid lines following the same color code as in A). The fully shaded shapes are the distance distributions derived from mtsslWizard. The structure assigned to the distribution is shown next to it. Middle panel: (**D**) Background corrected PELDOR time traces for ${\rm{P}}_{\rm{1}}^{{\rm{U18}}}$ in the absence (green line) and presence of 0.4 mM (light blue line) and 40 mM (blue line) Gdm^+^. The fits are overlaid as black dotted line. The modulation depths are indicated in percentages. (**E** and **F**) The distance distributions corresponding to the time traces in (D) shown as solid lines following the same color code as in (D). The fully shaded shapes are the distance distributions derived from mtsslWizard. The structure assigned to the distribution is shown next to it. Bottom panel: (**G**) Background corrected PELDOR time traces for the 1:1 mixture of ${\rm{P}}_{\rm{1}}^{{\rm{U18}}}$ and ${\rm{P}}_{\rm{2}}^{{\rm{U14}}}$ in the absence (grey line) and presence of 40 mM (black line) Gdm^+^. The fits are overlaid as grey or black dotted line, respectively. The modulation depths are indicated in percentages. (**H** and **I**) The distance distributions corresponding to the time traces in (**G**) shown as solid lines following the same color code as in (**G**). The fully shaded shapes are the distance distributions derived from mtsslWizard. The structure assigned to the distribution is shown next to it. Measurements were conducted on 25 μM RNA solution in 10 mM HEPES, 10 mM MgCl_2_, 10 mM KCl (pH 7.5) in D_2_O plus 20% ethylene glycol-d_6_ (v/v). The samples were annealed by incubating at 95°C for 5 min and subsequent addition on ice in absence (–Gdm^+^), in presence of 0.4 mM Gdm^+^ (+Gdm^+^) or 40 mM Gdm^+^ (++Gdm^+^) as denoted in the Figure, respectively.

In order to test whether the kissing hairpin homodimers found in the crystals are also formed in solution, PELDOR measurements were performed on the isolated hairpins **P1** and **P2** in the absence and presence of Gdm^+^. For both hairpins, the label site was chosen such that the wild-type sequence has a uridine at the respective position in order to minimize perturbations, and such that the kissing hairpin dimers yield label-to-label distances that fall within the PELDOR range. These theoretically distances were predicted by *in silico* spin labeling of the respective crystal structures (10,11) using the program MtsslWizard (32–34). This led to the constructs ${\rm{P}}_{\rm{1}}^{{\rm{U18}}}$ and ${\rm{P}}_{\rm{2}}^{{\rm{U14}}}$, where U18 and U14 are the labeled bases, respectively (see Figure 1A). For the **d**${{\bf \dot{U}}}$-label used here it has been shown that it is rigid and yields orientation selective PELDOR data (30). These can either be analyzed or the orientation selectivity can be removed by summing up PELDOR time traces recorded at various positions on the nitroxides EPR spectrum (30,48,52–55). The latter way was chosen here to ease analysis. The summed and background corrected PELDOR time traces for ${\rm{P}}_{\rm{1}}^{{\rm{U18}}}$ and ${\rm{P}}_{\rm{2}}^{{\rm{U14}}}$ are shown in Figure 3A-I together with the corresponding distance distributions. The original time traces and the data validation plots are collected in the SI.

The PELDOR time trace for ${\rm{P}}_{\rm{2}}^{{\rm{U14}}}$ displays in the absence of Gdm^+^ a modulation depth of 8% (Figure 3A). Since each hairpin ${\rm{P}}_{\rm{2}}^{{\rm{U14}}}$ carries only one label a modulation depth can only arise from such structures that are dimers. Higher order oligomers are unlikely for RNA. If all ${\rm{P}}_{\rm{2}}^{{\rm{U14}}}$ would form dimers one would expect under the used measurement conditions a modulation depth of ∼35%. Thus, only ∼23% of ${\rm{P}}_{\rm{2}}^{{\rm{U14}}}$ form some sort of dimer structure. Which dimer structure cannot be extracted because the corresponding distance distribution is broad indicating a wide distribution of structures (Figure 3B). Adding 0.4 mM Gdm^+^, which corresponds to the 2- to 8-fold of the *K*_D_ of the full-length construct (12), led to an increase in the modulation depth to 19% implying that Gdm^+^ induces the formation of more dimers, which constitute now 54% of the solution (Figure 3A). In addition to the increase in modulation depth, the time trace shows now also a pronounced oscillation, which transforms into a sharp peak at 3.7 nm in the corresponding distance distribution. This distance fits nicely to the distance one obtains from the kissing hairpin dimer found in the crystal structure of ${\rm{P}}_{\rm{2}}^{{\rm{U14}}}$ after modelling the label into the structure (Figure 3C). Thus, the addition of Gdm^+^ converted the mostly single stranded structures to a large extent into the kissing hairpin dimer ${\rm{P}}_{\rm{2}}^{{\rm{U14}}}\ |$${\rm{P}}_{\rm{2}}^{{\rm{U14}}}$.

In contrast, hairpin ${\rm{P}}_{\rm{1}}^{{\rm{U18}}}$ yields already in the absence of Gdm^+^ a modulation depth of 27% implying that in this case ∼78% of the RNA strands form dimers even in the absence of Gdm^+^ (Figure 3D). Since the time trace is modulated, the corresponding distance distribution reveals a defined peak, which has its maximum at 4.8 nm (Figure 3E). This distance does not fit to the kissing hairpin structure found for the homodimer **P1 $|$ P1** in the crystal but perfectly to an A-form RNA duplex formed from two ${\rm{P}}_{\rm{1}}^{{\rm{U18}}}$ strands. This duplex formation in the absence of Gdm^+^ was also found in crystallization attempts (56). Adding Gdm^+^ in concentrations of 0.4 mM and of 40 mM changes the modulation depth to 34% and 32%, respectively, and induces the formation of a different dimer structure, now with a distance of 4.1 nm between both labels. A close inspection of the distance distribution corresponding to 0.4 mM Gdm^+^ indicates a superposition of the peaks in the absence and presence of 40 mM Gdm^+^ (68% duplex versus 32% kissing hairpin, see Supplementary Figure S13). The new peak at 4.1 nm fits to the distance one can infer from the kissing hairpin dimer found in the crystal structure (Figure 3F). Thus, PELDOR confirms that **P1** and **P2** adopt also in solution the kissing hairpin dimers in the presence of Gdm^+^. Furthermore, it revealed that truncated **P1** adopts a duplex structure in the absence of Gdm^+^, while the truncated **P2** assumes various different structures. Interestingly, for ${\rm{P}}_{\rm{2}}^{{\rm{U14}}}$ a Gdm^+^ concentration of 0.4 mM was sufficient to dominantly form the kissing hairpin dimer, which does also not change upon adding more Gdm^+^ (see Supplementary Figure S14), whereas a Gdm^+^ concentration of 40 mM is needed for ${\rm{P}}_{\rm{1}}^{{\rm{U18}}}$. This may be related to ${\rm{P}}_{\rm{1}}^{{\rm{U18}}}$ having three additional G-C base-pairs, which stabilizes the P1:P1 duplex and requires a higher Gdm^+^ concentration to shift the equilibrium towards the kissing hairpin dimer.

Mixing both hairpins, ${\rm{P}}_{\rm{1}}^{{\rm{U18}}}$ and ${\rm{P}}_{\rm{2}}^{{\rm{U14}}}$ in a 1:1-ratio yields the PELDOR time traces and distance distributions in Figure 3G-I. In the absence of Gdm^+^, the PELDOR time trace shows a modulation depth of ∼21% but with no clear oscillation and a broad distance distribution between 3 and 6 nm. Thus, the mixture in the absence of Gdm^+^ behaves like the sum of the isolated hairpins ${\rm{P}}_{\rm{1}}^{{\rm{U18}}}$ and ${\rm{P}}_{\rm{2}}^{{\rm{U14}}}$. Adding Gdm^+^ causes an increase in the modulation depth to ∼30%, indicating that now 86% of the RNA form dimers, and the occurrence of an oscillation in the time trace. The corresponding distance distribution shows a peak at about 4.0 nm, which might either result from a superposition of the homodimers of ${\rm{P}}_{\rm{1}}^{{\rm{U18}}}\ |\ {\rm{P}}_{\rm{1}}^{{\rm{U18}}}$and ${\rm{P}}_{\rm{2}}^{{\rm{U14}}}\ |\ {\rm{P}}_{\rm{2}}^{{\rm{U14}}}$ and/or of the heterodimer ${\rm{P}}_{\rm{1}}^{{\rm{U18}}}$ | ${\rm{P}}_{\rm{2}}^{{\rm{U14}}}$. Which of these cases is present cannot be distinguished because the *in silico* derived distances are with 4.1, 3.7 and 3.5 nm too close (Figure 3I).

**Figure 4.**
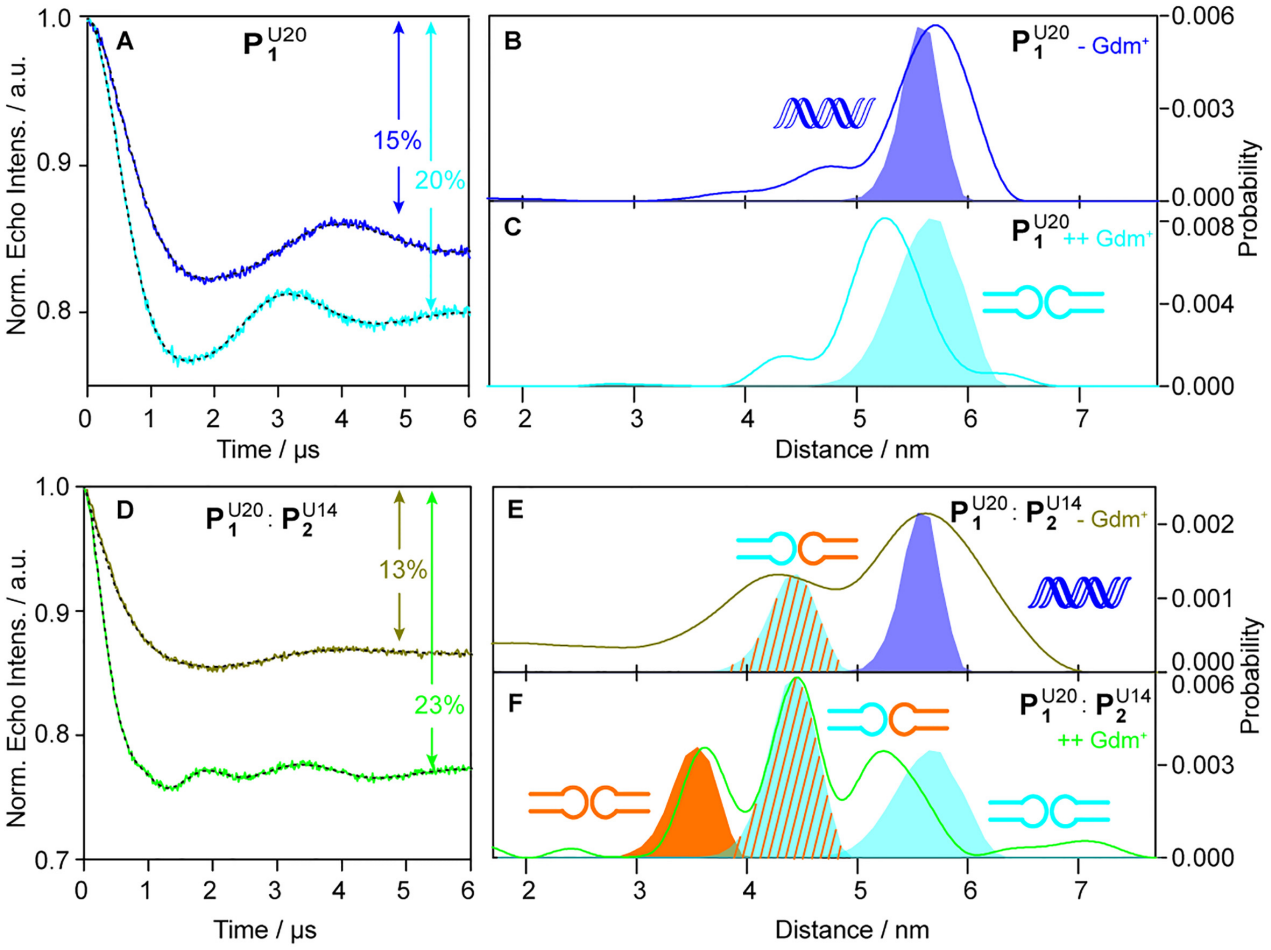
PELDOR time traces, distance distributions and assigned structures for ${\rm{P}}_{\rm{2}}^{{\rm{U14}}}$ and ${\rm{P}}_{\rm{1}}^{{\rm{U20}}}$. Top panel: (**A**) Background corrected PELDOR time traces for ${\rm{P}}_{\rm{1}}^{{\rm{U20}}}$ in the absence (blue line) and presence (cyan line) of Gdm^+^. The fits are overlaid as black dotted line. The modulation depths are indicated in percentages. (**B** and **C**) The distance distributions corresponding to the time traces in A) shown as solid lines following the same color code as in A). The fully shaded shapes are the distance distributions derived from mtsslWizard. The structure assigned to the distribution is shown next to it. Bottom panel: (**D**) Background corrected PELDOR time traces for the 1:1 mixture of ${\rm{P}}_{\rm{1}}^{{\rm{U20}}}$and ${\rm{P}}_{\rm{2}}^{{\rm{U14}}}$ in the absence (olive line) and presence (green line) of 40 mM Gdm^+^. The fits are overlaid as black dotted line. The modulation depths are indicated in percentages. (**E** and **F**) The distance distributions corresponding to the time traces in (D) shown as solid lines following the same color code as in (D). The fully shaded shapes are the distance distributions derived from mtsslWizard. The structure assigned to the distribution is shown next to it. Measurements were conducted on 25 μM RNA samples (10 mM HEPES, 10 mM MgCl_2_, 10 mM KCl; pH 7.5) in D_2_O plus 20% ethylene glycol-d_6_ (v/v). The samples were annealed by incubating at 95°C for 5 min and subsequent cooling on ice in absence (–Gdm^+^) or presence of 40 mM Gdm^+^ (++Gdm^+^) as denoted in the Figure, respectively.

For a better separation between the distance distributions of the homo- and heterodimers a new spin label position was chosen for **P1**, namely U20. Using this label site, the new homodimer ${\rm{P}}_{\rm{1}}^{{\rm{U20}}}\ |\ {\rm{P}}_{\rm{1}}^{{\rm{U20}}}$ has an *in silico* predicted label-to-label distance of 5.6 nm and the predicted distance for the new heterodimer ${\rm{P}}_{\rm{1}}^{{\rm{U20}}}\ |\ $${\rm{P}}_{\rm{2}}^{{\rm{U14}}}$ moves to 4.5 nm whereas the distance for the homodimer ${\rm{P}}_{\rm{2}}^{{\rm{U14}}}\ |\ {\rm{P}}_{\rm{2}}^{{\rm{U14}}}$ stays at 3.5 nm (Figure 4). Thus, all three dimers are separated by 1 or 2 nm from each other, which can be easily distinguished by PELDOR. The PELDOR measurements on the isolated ${\rm{P}}_{\rm{1}}^{{\rm{U20}}}$ reveals similar results as for ${\rm{P}}_{\rm{1}}^{{\rm{U18}}}$. In the absence and in the presence of Gdm^+^ defined distance distributions are observed (Figure 4A–C) with distances of 5.7 and 5.2 nm, respectively. Taken together with the increase in dimer population from 43 to 57% this indicates again a structural change upon adding Gdm^+^. However, for the new spin labeling position, the theoretically obtained distances for the duplex (Figure 4B) and kissing hairpin dimer (Figure 4C) are both ∼5.6 nm making an assignment impossible. Yet, the distance shift after adding Gdm^+^ makes it likely, that the duplex is present in the absence of Gdm^+^ and that it is converted in to the kissing hairpin homodimer in the presence of 40 mM Gdm^+^, in agreement with the data for ${\rm{P}}_{\rm{1}}^{{\rm{U18}}}$.

Nevertheless, the idea for the new label site in **P1** was to distinguish kissing hairpin homodimer from heterodimer. Therefore, ${\rm{P}}_{\rm{1}}^{{\rm{U20}}}$ and ${\rm{P}}_{\rm{2}}^{{\rm{U14}}}$were mixed in a ratio of 1:1 and PELDOR measurements were conducted in the absence and presence of Gdm^+^ on this mixture (Figure 4D-F). In the absence of Gdm^+^, the PELDOR time trace shows a modulation depth of 13% (36% dimer) and a corresponding distance distribution with two peaks at 4.2 nm and 5.6 nm. The long distance is assigned to the ${\rm{P}}_{\rm{1}}^{{\rm{U20}}}$ forming probably A-form RNA duplexes, whereas the short one is assigned to the kissing hairpin heterodimer ${\rm{P}}_{\rm{1}}^{{\rm{U20}}}$ | ${\rm{P}}_{\rm{2}}^{{\rm{U14}}}$. The latter assignment is based on the agreement with the mtsslWizard prediction for this structure. Thus, it seems that in the mixture the heterodimer is already formed in the absence of Gdm^+^. Adding 40 mM Gdm^+^ leads to a clear result: The modulation depth increases to 23%, corresponding to 66% dimers, and three distinct peaks are observed at 3.6 nm, 4.4 nm and 5.2 nm. The short and the long distance match the distances for the homodimers ${\rm{P}}_{\rm{1}}^{{\rm{U20}}}|\ {\rm{P}}_{\rm{1}}^{{\rm{U20}}}$and ${\rm{P}}_{\rm{2}}^{{\rm{U14}}}$ | ${\rm{P}}_{\rm{2}}^{{\rm{U14}}}$, respectively, and the peak at 4.5 nm fits perfectly to the predicted distance of the heterodimer ${\rm{P}}_{\rm{1}}^{{\rm{U20}}}$ | ${\rm{P}}_{\rm{2}}^{{\rm{U14}}}$. In addition, the distance distribution reveals within the error (Supplementary Figure S8) and based on the amplitude and area of the peaks a 1:2:1 ratio for ${\rm{P}}_{\rm{1}}^{{\rm{U20}}}\ |\ {\rm{P}}_{\rm{1}}^{{\rm{U20}}}$, ${\rm{P}}_{\rm{1}}^{{\rm{U20}}}\ |\ {\rm{P}}_{\rm{2}}^{{\rm{U14}}}$and ${\rm{P}}_{\rm{2}}^{{\rm{U14}}}$ | ${\rm{P}}_{\rm{2}}^{{\rm{U14}}}$, respectively (Supplementary Figure S15, Supplementary Tables S8 and S9). Thus, there is only a statistical distribution of the dimers and no indication of a preferred dimer interaction between **P1** and **P2** over the homodimer interaction in the presence of Gdm^+^.

## DISCUSSION

Here, it could be shown that the hairpins **P1** and **P2** of the guanidine-II riboswitch form in the absence of Gdm^+^ different structures, with **P1** favoring an A-form RNA duplex structure. Adding Gdm^+^ leads in both cases to the formation of the kissing loop homodimers that were also found in the crystals but in the case of **P1** a larger excess of Gdm^+^ is needed for full conversion. Importantly, the mixture of both hairpins does clearly show formation of the expected **P1 $|$ P2** kissing hairpin heterodimer but only in a statistical mixture with the homodimers. Nevertheless, the observation of the heterodimer does support the proposed mechanistic model. In an earlier study, the binding of Gdm^+^ to construct with linked **P1** and **P2** stem loops was monitored via quenching of the fluorescence of 2-aminopurine (2-Ap at A10 in **P2**). Indeed, fluorescence quenching was observed upon addition of Gdm^+^ (13). However, any structural change that leads to more stacking or more shielding from water would lead to quenching of this fluorescence. Even if the hairpins would interact, this experiment cannot distinguish between an interstrand **P2 $|$ P2** or intrastrand **P1 $|$ P2** interaction. Whether the full-length guanidine-II riboswitch does indeed show such an intrastrand **P1 $|$ P2** interaction is currently under study.

## Supplementary Material

gkaa703_Supplemental_File
