# Supplementary material for: Do the P1 and P2 hairpins of the Guanidine-II riboswitch interact?
Source: Nucleic Acids Res. 2020 Aug 28;48(18):10518–26. doi: 10.1093/nar/gkaa703 (PMC7544219; doi:10.1093/nar/gkaa703)
Supplement: gkaa703_Supplemental_File [file gkaa703_supplemental_file.docx]

Do the P1 and P2 hairpins of the Guanidine-II Riboswitch interact?

Christine Wuebben^1^, Maria F. Vicino^1^, Marcel Mueller^1^, Olav Schiemann^1*^

### DOI: 10.1002/anie.2016XXXXX

Supplemental Material

### **Table of Contents**

1. **RNA Characterization 3**
   1. RNA Sequences 3
   2. Determination of RNA Concentrations 3
   3. HPLC Analysis of the Spin Labeled RNA Constructs 3
   4. Yields 3
   5. LCMS Analysis of the Spin Labeled RNA Constructs 4
2. **CW EPR 4**
   1. CW EPR Concentrations and Spin Counting 5
3. **PELDOR 5**
   1. PELDOR Raw Data 6
   2. PELDOR Data Analysis of the Summed Time Traces 7
   3. PELDOR Data Validation 9
4. **Characterization of the Spin Label Influence 10**
   1. CD Spectroscopy 10
   2. T_m_ Measurements 10
   3. Native PAGEs 10
5. **PELDOR Derived Distance Distribution Analysis 12**
   1. Distance Distribution Fit of $\text{P}_{\text{1}}^{\text{U18}}$ 12
   2. PELDOR on $\text{P}_{\text{2}}^{\text{U14}}$ in Presence of 0.4 mM Gdm^+^ and 100 mM Gdm^+^ 12

5.3 Statistics of the Distance Distribution of $\text{P}_{\text{1}}^{\text{U20}} \mathbf{|} \text{P}_{\text{2}}^{\text{U14}}$ in Presence of Gdm^+^ 12

1. RNA Characterization

1.1 RNA Sequences

The RNA constructs were purchased from *metabion* *international AG (*Planegg, Germany*)* with the denoted sequences in Table S1.

**Table S1.** List of the RNA constructs.^[a]^

| Construct | Sequence 5’ to 3’ |
| --- | --- |
| $\text{P}_{\text{1}}^{\text{unmod}}$ | GGA AGC GGG ACG ACC CGU UUU C |
| $\text{P}_{\text{1}}^{\text{U18}}$ | GGA AGC GGG ACG ACC CG**X** UUU C |
| $\text{P}_{\text{1}}^{\text{U20}}$ | GGA AGC GGG ACG ACC CGU U**X**U C |
| $\text{P}_{\text{2}}^{\text{unmod}}$ | GCG GGG ACG ACC CUG C |
| $\text{P}_{\text{2}}^{\text{U14}}$ | GCG GGG ACG ACC C**X**G C |

[a] The marked **X** is 5-ethynyl-2’-deoxy-uridine and the spin labeling position.

1.2 Determination of RNA Concentrations

Nucleic acid concentrations were determined by measuring the absorbance of ultraviolet light using a NanoDrop Spectrophotometer (*Thermo Scientific*) and the Lambert-Beer law. The extinction coefficients used are listed in Table S2.

**Table S2.** Extinction coefficients as provided by *metabion* *international AG*.

| Construct | Extinction coefficient / Lmol^-1^cm^-1^ |
| --- | --- |
| $\text{P}_{\text{1}}^{\text{unmod}}$, $\text{P}_{\text{1}}^{\text{U18}}$, $\text{P}_{\text{1}}^{\text{U20}}$ | 216000 |
| $\text{P}_{\text{2}}^{\text{unmod}}$,$\text{P}_{\text{2}}^{\text{U14}}$ | 147000 |

1.3 HPLC analysis of the Spin Labeled RNA Constructs

**
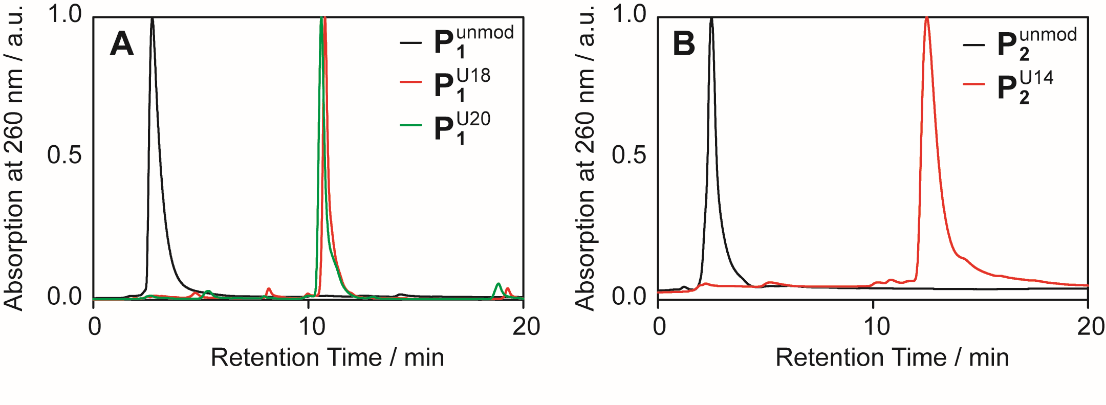
**

**Figure S1.** HPLC analysis showing the UV-trace at 260 nm of (A) $\text{P}_{\text{1}}^{\text{unmod}}$, $\text{P}_{\text{1}}^{\text{U18}}$ and $\text{P}_{\text{1}}^{\text{U20}}$, (B) $\text{P}_{\text{2}}^{\text{unmod}}$ and $\text{P}_{\text{2}}^{\text{U14}}$.

1.4 Yields

**Table S3.** Final yields of the labeled RNA constructs.

| Construct | Yield / % |
| --- | --- |
| $\text{P}_{\text{1}}^{\text{U18}}$ | 60 |
| $\text{P}_{\text{1}}^{\text{U20}}$ | 72 |
| $\text{P}_{\text{2}}^{\text{U14}}$ | 60 |

1.5 LCMS Analysis of the Spin Labeled RNA Constructs

**
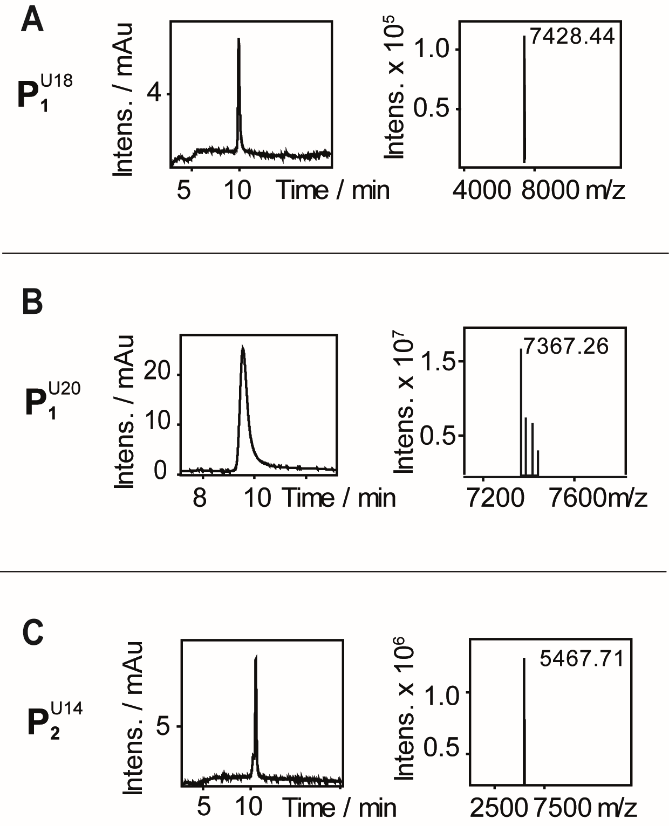
**

**Figure S2.** LCMS Analysis of the spin labeled RNA constructs. The left panel shows the UV trace at 260 nm and the right panel the deconvoluted ESI^-^ data.

**Table S4.** Calculated and observed mass of the labeled RNA constructs.

| Construct | M_calcd._^[a]^ | M_observed_^[b]^ |
| --- | --- | --- |
| $\text{P}_{\text{1}}^{\text{U18}}$ | 7365.23 | 7428.44^[c]^ |
| $\text{P}_{\text{1}}^{\text{U20}}$ | 7365.23 | 7367.26 |
| $\text{P}_{\text{2}}^{\text{U14}}$ | 5443.10 | 5467.71^[d]^ |

[a] The values are calculated using the Mongo Oligo calculator v2.08.

[b] Main peak in deconvoluted spectra.

[c] M_found_ is assigned to [M+^23^Na+^39^K]^+.^

[d] M_found_ is assigned to [M+^23^Na]^+.^

2. CW EPR


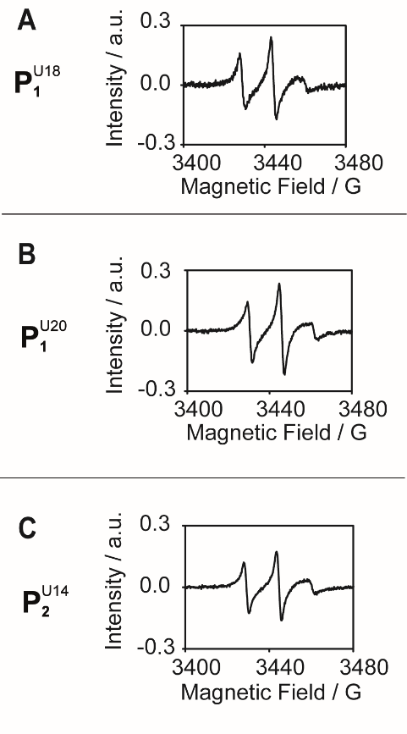


**Figure S3.** CW X-band EPR spectra of (A) $\text{P}_{\text{1}}^{\text{U18}}$, (B) $\text{P}_{\text{1}}^{\text{U20}}$ and (C) $\text{P}_{\text{2}}^{\text{U14}}$.

2.1 CW EPR Concentrations and Spin Counting

**Table S5.** Spin concentrations.

| Construct |  | c / µM |
| --- | --- | --- |
| $\text{P}_{\text{1}}^{\text{U18}}$ | set | 25 |
|  | measured | 26 |
| $\text{P}_{\text{1}}^{\text{U20}}$ | set | 25 |
|  | measured | 25 |
| $\text{P}_{\text{2}}^{\text{U14}}$ | set | 25 |
|  | measured | 24 |

**3. PELDOR**
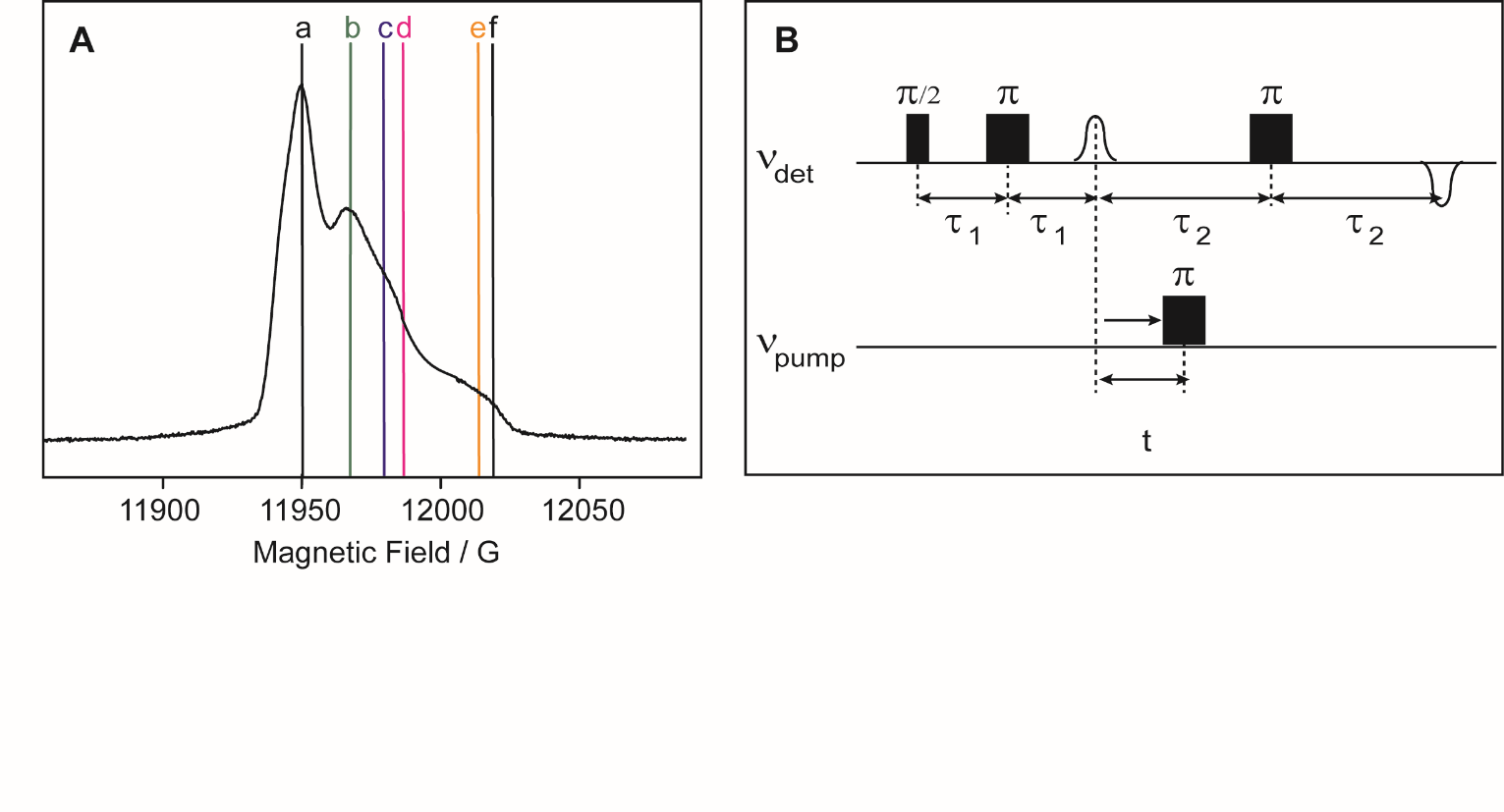


**Figure S4.** (A) Echo detected Q-band field sweep spectrum with positions of the pump and probe pulses indicated (the colour code is the same as in Table S6 and Figure S5). (B) PELDOR pulse sequence.

**Table S6.** List of the Frequency offsets.^[a]^

| Pump position | Detection position | Frequency offset / MHz |
| --- | --- | --- |
| a | b | 60 |
| a | c | 80 |
| a | d | 100 |
| b | e | 120 |
| a | f | 180 |

[a] The color code corresponds to the measurements in Figure S5.

**3.2 PELDOR Raw Data**

**
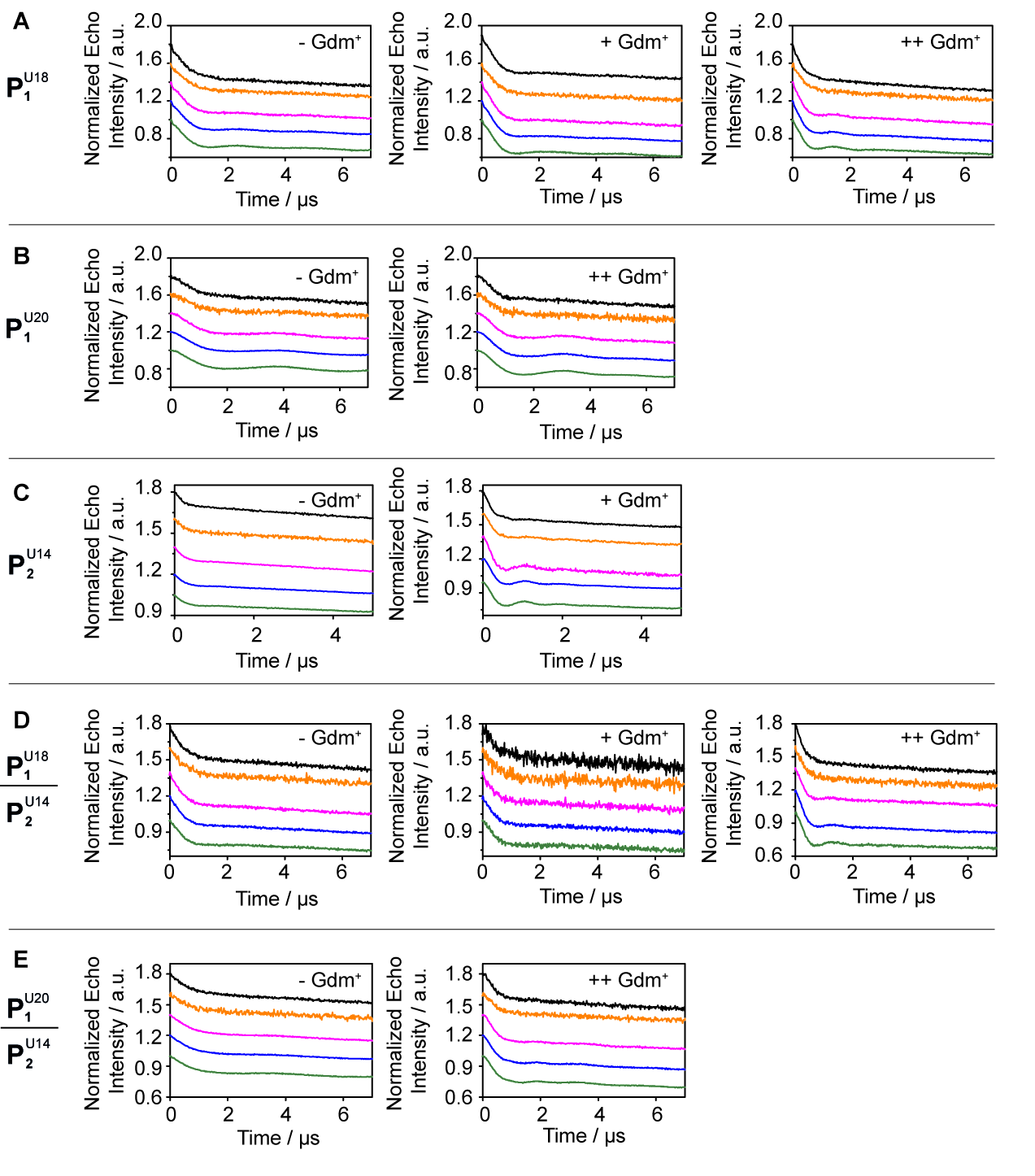
Figure S5.** Raw Data of the offset measurements in absence and presence of Gdm^+^. (A) $\text{P}_{\text{1}}^{\text{U18}}$, (B) $\text{P}_{\text{1}}^{\text{U20}}$, (C) $\text{P}_{\text{2}}^{\text{U14}}$, (D) $\text{P}_{\text{1}}^{\text{U18}}$ mixed 1:1 with $\text{P}_{\text{2}}^{\text{U14}}$ and (E) $\text{P}_{\text{1}}^{\text{U20}}$ mixed 1:1 with $\text{P}_{\text{2}}^{\text{U14}}$.


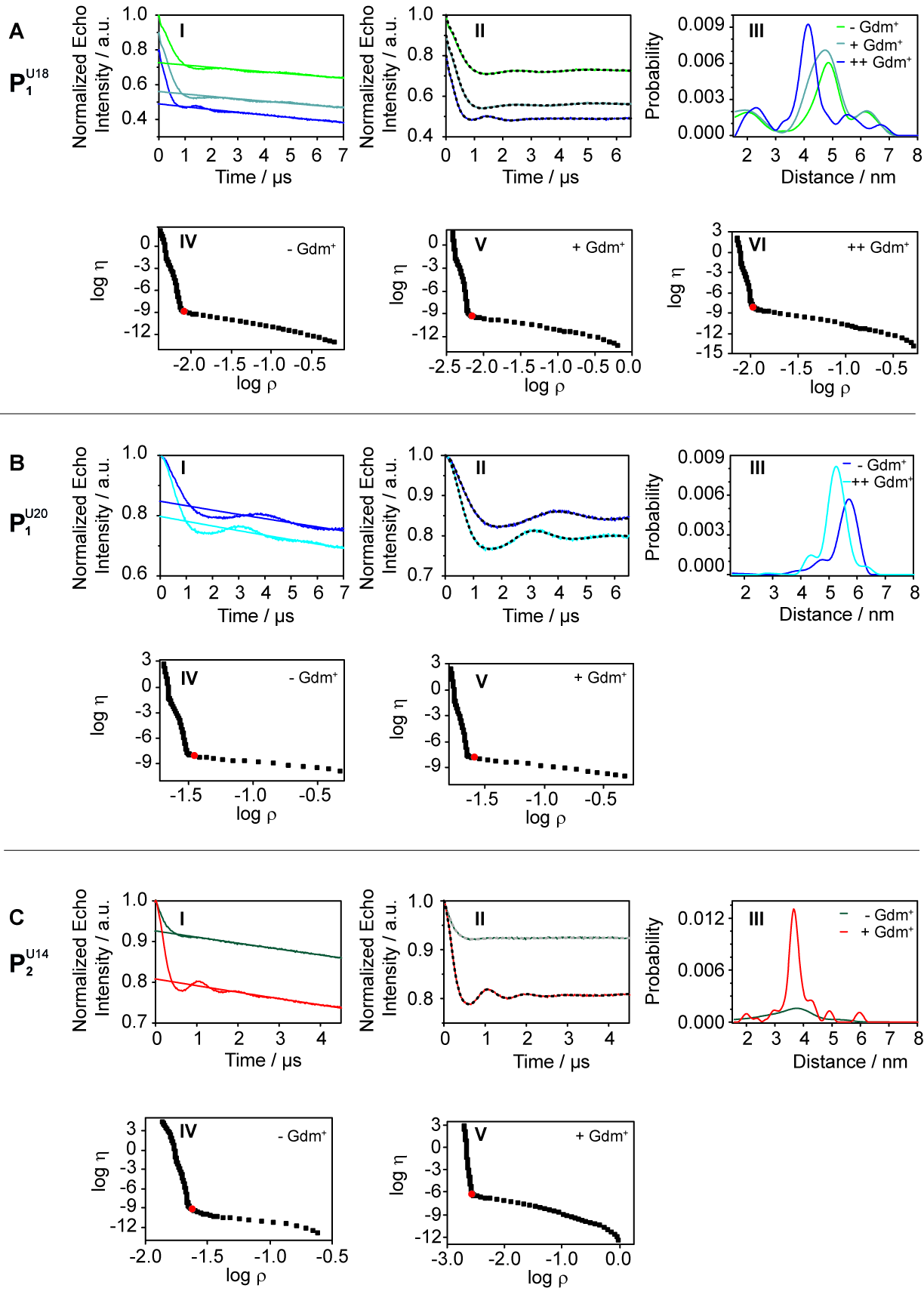
3.2 PELDOR Data Analysis of the Summed Time Traces

**Figure S6.** Data Analysis of the summed PELDOR time traces in the absence and presence of Gdm^+^ for (A) $\text{P}_{\text{1}}^{\text{U18}}$, (B) $\text{P}_{\text{1}}^{\text{U20}}$ and (C) $\text{P}_{\text{2}}^{\text{U14}}$. (I) Uncorrected PELDOR time traces with the intermolecular background function. (II) Background corrected PELDOR time traces with overlaid fit (dotted black line). (III) PELDOR-derived distance distributions. (IV)-(VI): The corresponding L-curves.


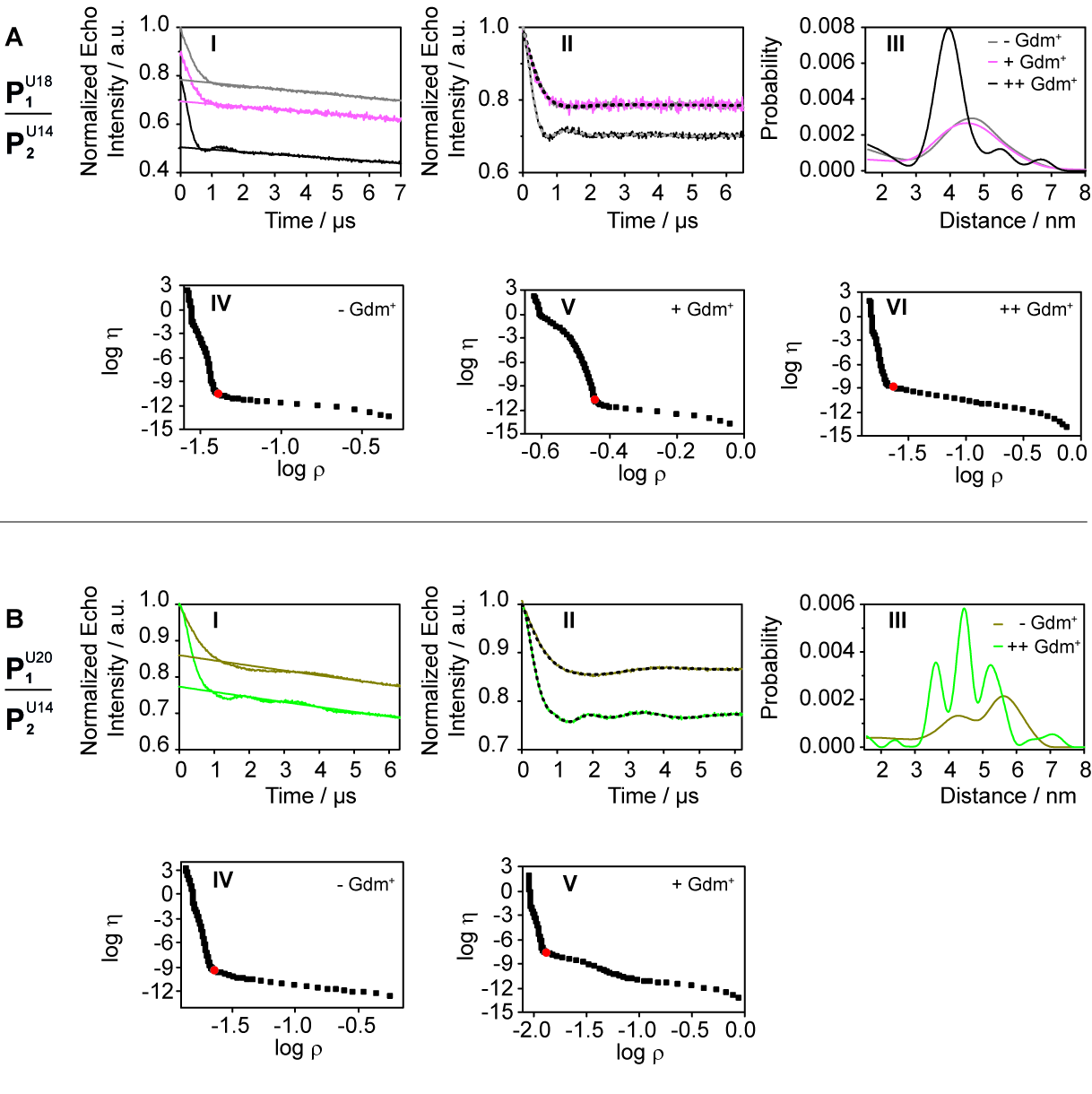


**Figure S7.** Data Analysis with DeerAnalysis (1) of the summed PELDOR time traces in absence and presence of Gdm^+^. (A) $\text{P}_{\text{1}}^{\text{U18}}$ mixed 1:1 with $\text{P}_{\text{2}}^{\text{U14}}$ and (B) $\text{P}_{\text{1}}^{\text{U20}}$ mixed 1:1 with $\text{P}_{\text{2}}^{\text{U14}}$. (I) Uncorrected PELDOR time traces with the intermolecular background function. (II) Background corrected PELDOR time traces with fit overlaid (dotted black line). (III) PELDOR-derived distance distributions. (IV)-(VI), the corresponding L-curves.

3.3. PELDOR Data Validation

**
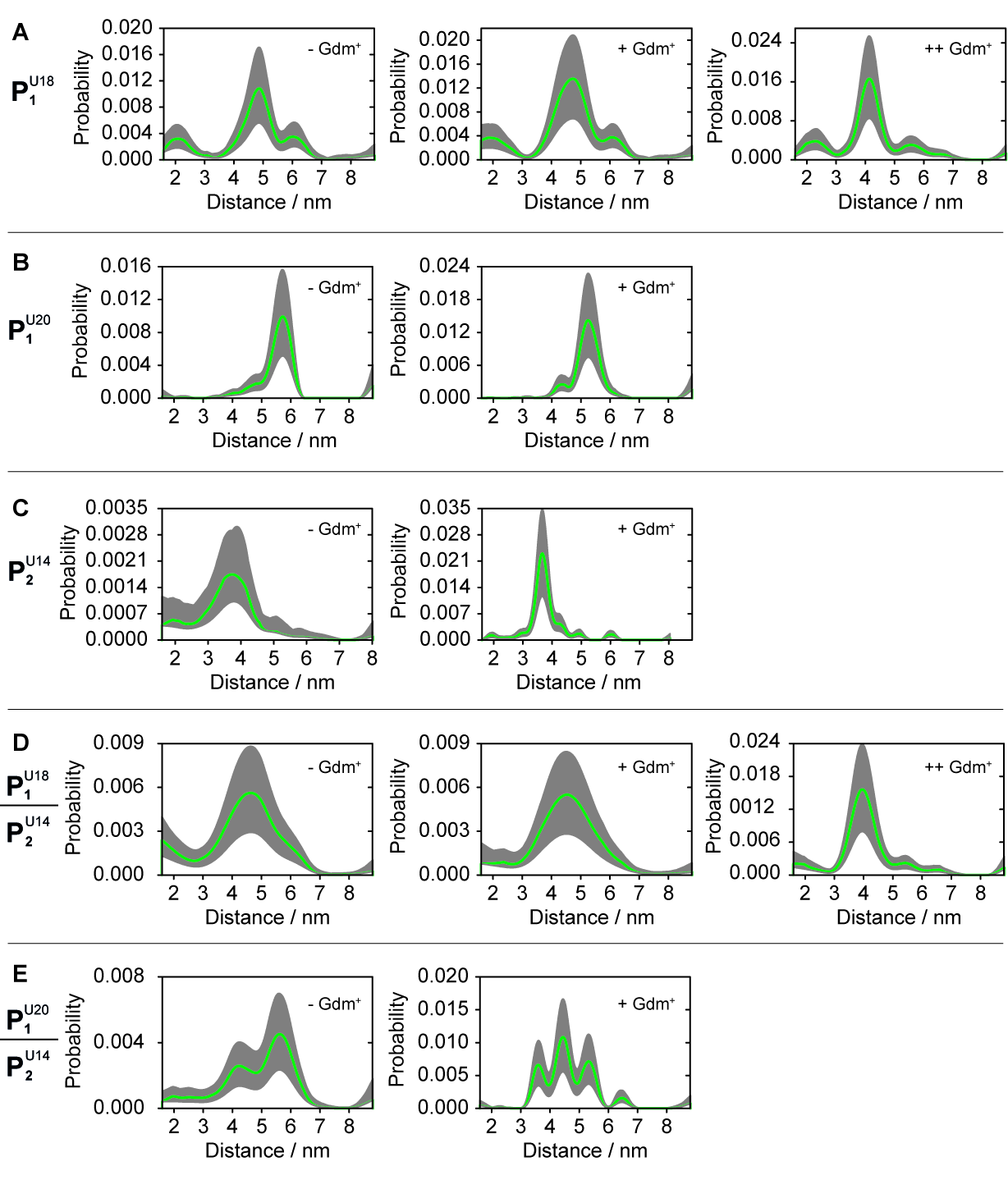
**

**Figure S8.** Data Validation of the summed PELDOR time traces from Figure S6 and Figure S7. (A) $\text{P}_{\text{1}}^{\text{U18}}$, (B) $\text{P}_{\text{1}}^{\text{U20}}$, (C) $\text{P}_{\text{2}}^{\text{U14}}$, (D) $\text{P}_{\text{1}}^{\text{U18}}$ mixed 1:1 with $\text{P}_{\text{2}}^{\text{U14}}$ and (E) $\text{P}_{\text{1}}^{\text{U20}}$ mixed 1:1 with $\text{P}_{\text{2}}^{\text{U14}}$.

**4. Characterization of the Spin Label Influence**


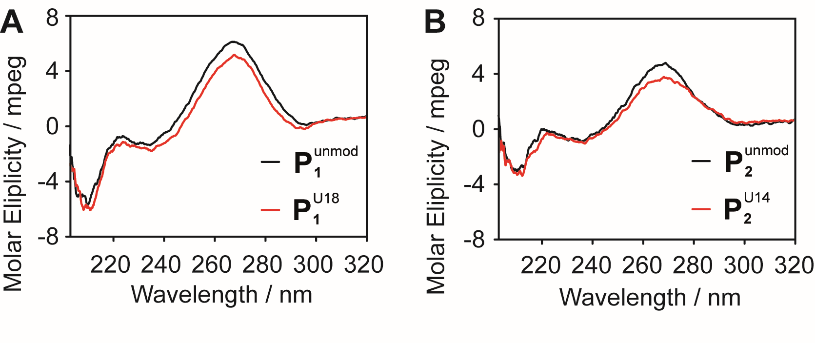
4.1 CD Spectroscopy

**Figure S9.** CD spectra in the presence of 40 mM Gdm^+^. (A) $\text{P}_{\text{1}}^{\text{unmod}}$and $\text{P}_{\text{1}}^{\text{U18}}$. (B) $\text{P}_{\text{2}}^{\text{unmod}}$ and $\text{P}_{\text{2}}^{\text{U14}}$. The labeling position of $\text{P}_{\text{1}}^{\text{U20}}$ was chosen two base pairs below of $\text{P}_{\text{1}}^{\text{U18}}$ and its influence on the structure was judged as being comparable.

4.2 Tm Measurements


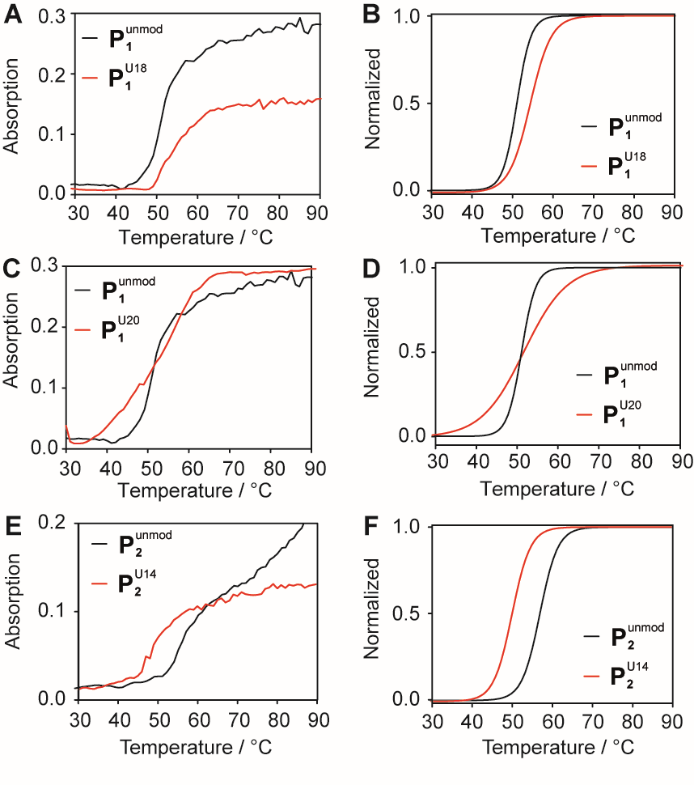
The UV-VIS data (Figure S4 A,C,E) were baseline corrected, normalized, and the curves fitted with the *Boltzmann function* in *OrginLab* (Figure S10 B,D,F). The derived parameter x_0_ was taken as the T_m_ value.

**Figure S10.** UV-VIS spectra recorded at 260 nm in the presence of 40 mM Gdm^+^. The left panel shows the raw data and the right panel the baseline corrected and fitted data. (A) and (B) $\text{P}_{\text{1}}^{\text{unmod}}$and $\text{P}_{\text{1}}^{\text{U18}}$; (C) and (D) $\text{P}_{\text{1}}^{\text{unmod}}$and $\text{P}_{\text{1}}^{\text{U20}}$; (E) and (F) $\text{P}_{\text{2}}^{\text{unmod}}$ and $\text{P}_{\text{2}}^{\text{U14}}$.

**Table S7.** Tm values.

| Construct | Tm value / °C |
| --- | --- |
| $\text{P}_{\text{1}}^{\text{unmod}}$ | 51.0 |
| $\text{P}_{\text{1}}^{\text{U18}}$ | 54.4 |
| $\text{P}_{\text{1}}^{\text{U20}}$ | 51.7 |
| $\text{P}_{\text{2}}^{\text{unmod}}$ | 56.9 |
| $\text{P}_{\text{2}}^{\text{U14}}$ | 50.0 |

4.3 Native PAGEs

Native PAGEs were run to investigate whether conformational changes can be detected for **P1** and **P2** upon adding Gdm^+^ and whether there are differences between unmodified and labeled RNA (**Figure S11** and **Figure S12**). Standard staining procedures with ethidium bromide did not yield analysable bands, probably because the short stem structures prevented intercalation, but using GelRed instead provided bands. The results show that the gel shifts are in all cases comparable for the unmodified and labeled RNA. Performing the native PAGE in the absence of Gdm^+^ in the running buffer show a band shift upon adding Gdm^+^ during the annealing procedure (**Figure S11**). However, this band shift corresponds to a considerably faster migrating and thus smaller RNA construct, most likely the monomeric hairpins. This might indicate that Gdm^+^ is removed from the hairpins under the gel electrophoresis conditions. This is supported by the band-shift corresponding to a slower migrating construct, when Gdm+ is added to the running buffer (**Figure S12**), indicating that the kissing hairpin dimers do prevail under the gel-electrophoresis conditions. Thus, without Gdm^+^ in the running buffer and in absence of Gdm^+^ during the sample preparation, the duplex is the dominant conformation on the gel. Without Gdm^+^ in the running buffer and in presence of Gdm^+^ during the sample preparation, monomer hairpin is the dominant conformation on the gel. With Gdm^+^ in the running buffer and in presence of Gdm^+^ during the sample preparation, the kissing hairpin dimer is the dominant conformation on the gel. Nevertheless, and as also indicated by CD and UV-VIS spectroscopy, the spin label does not seem to alter the basic RNA fold and importantly, the conformation changes after adding Gdm^+^.


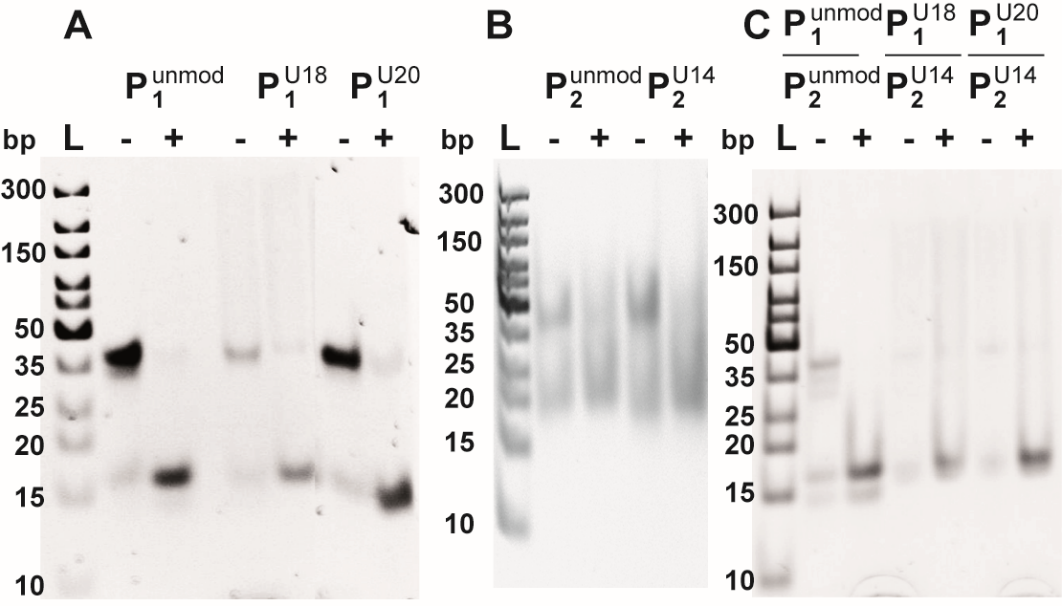


**Figure S11.** Native PAGE analysis in the absence of Gdm^+^ in the running buffer. (A) 15 % native PAGE in the absence (-) and presence (+) of Gdm^+^ for $\text{P}_{\text{1}}^{\text{unmod}}$, $\text{P}_{\text{1}}^{\text{U18}}$ and $\text{P}_{\text{1}}^{\text{U20}}$. (B) 15% native PAGE in the absence (-) and presence (+) of Gdm^+^ for $\text{P}_{\text{2}}^{\text{unmod}}$ and $\text{P}_{\text{2}}^{\text{U14}}$. (C) 15% native PAGE in the absence (-) and presence (+) of Gdm^+^ for $\text{P}_{\text{1}}^{\text{unmod}}$ mixed equivalently with$\text{P}_{\text{2}}^{\text{unmod}}$, $\text{P}_{\text{1}}^{\text{U18}}$ mixed equivalently with $\text{P}_{\text{2}}^{\text{U14}}$ and $\text{P}_{\text{1}}^{\text{U20}}$ mixed equivalently with $\text{P}_{\text{2}}^{\text{U14}}$. In each case, the RNA was annealed in 10 mM HEPES, 10 mM KCl and 10 mM MgCl_2_ (pH 7.5) in absence or presence of 40 mM Gdm^+^ by incubating at 95°C for 5 min and subsequently cooling on ice for 10 min. 89 mM Tris base, 89 mM boric acid, 25 mM NaCl was employed as running buffer. “L” depicts the GeneRuler ultra low range DNA ladder (ThermoFisher) with the base pair sizes on the left.


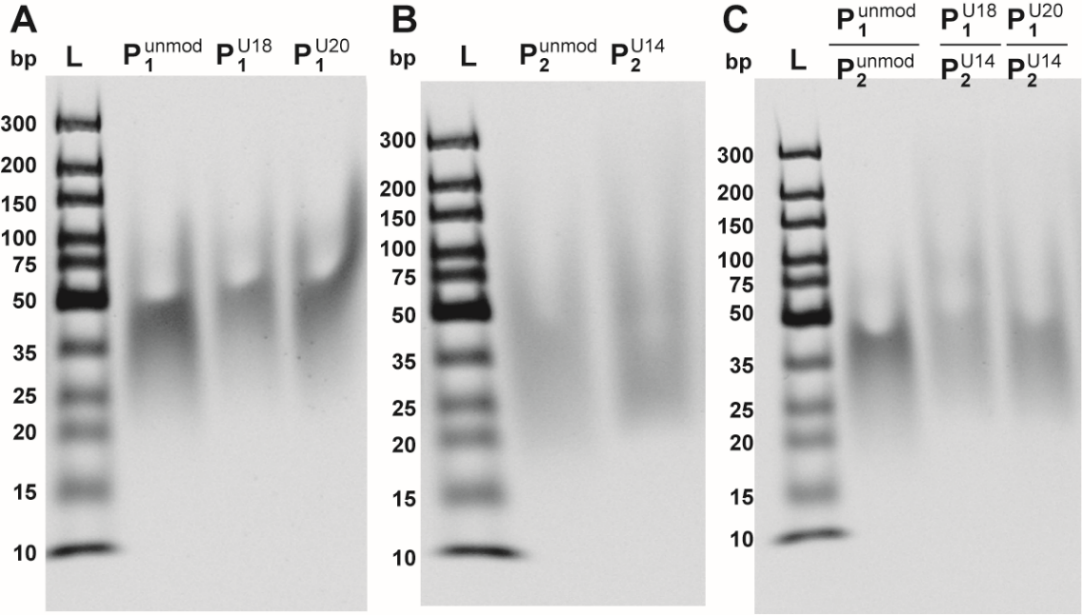


**Figure S12.** 15% Native PAGE in the presence of 40 mM Gdm^+^ in the running buffer. (A) $\text{P}_{\text{1}}^{\text{unmod}}$, $\text{P}_{\text{1}}^{\text{U18}}$ and $\text{P}_{\text{1}}^{\text{U20}}$; (B) $\text{P}_{\text{2}}^{\text{unmod}}$ and $\text{P}_{\text{2}}^{\text{U14}}$; (C) $\text{P}_{\text{1}}^{\text{unmod}}$ mixed equivalently with$\text{P}_{\text{2}}^{\text{unmod}}$, $\text{P}_{\text{1}}^{\text{U18}}$ mixed equivalently with $\text{P}_{\text{2}}^{\text{U14}}$ and $\text{P}_{\text{1}}^{\text{U20}}$ mixed equivalently with $\text{P}_{\text{2}}^{\text{U14}}$. In each case, the RNA was annealed in 10 mM HEPES, 10 mM KCl and 10 mM MgCl_2_ (pH 7.5) in presence of 40 mM Gdm^+^ by incubating at 95°C for 5 min and subsequently cooling on ice for 10 min. 40 mM Gdm^+^, 89 mM Tris Base, 89 mM Boric Acide, 25 mM NaCl was employed as running buffer. “L” depicts the GeneRuler ultra low range DNA ladder (ThermoFisher) with the base pair sizes on the left.

**5. PELDOR Derived Distance Distribution Analysis**

5.1 Distance Distribution Fit of $\text{P}_{\text{1}}^{\text{U18}}$


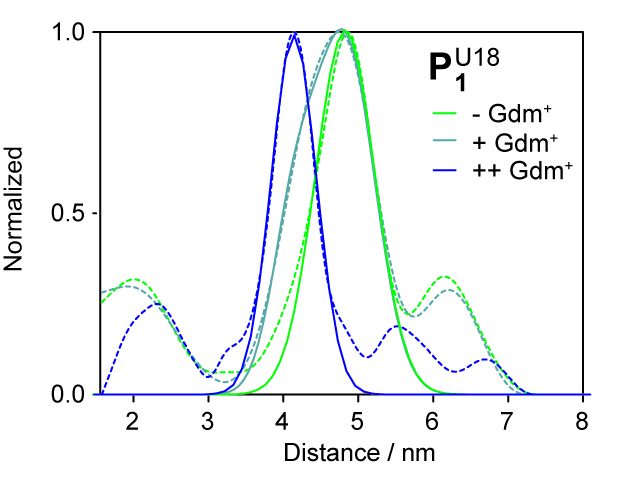
The distance distributions of $\text{P}_{\text{1}}^{\text{U18}}$ in absence (-Gdm^+^) and presence of 40 mM Gdm^+^ (++Gdm^+^) were fitted with Gaussian curves. The distribution for 0.4 mM Gdm^+^ could be reproduced by superimposing both Gaussians with the percentages 68% of the duplex (-Gdm^+^) and 32% of the kissing hairpin (++Gdm^+^), indicating that not an intermediate is formed.

**Figure S13.** Data Analysis of $\text{P}_{\text{1}}^{\text{U18}}$. The dashed lines are the measured distance distributions whereas the full lines show the Gauss-Fit.

5.2 PELDOR on $\text{P}_{\text{2}}^{\text{U14}}$ in Presence of 0.4mM Gdm^+^ and 100 mM Gdm^+^

PELDOR measurements in presence of 0.4 mM and 100 mM Gdm^+^ were conduct on $\text{P}_{\text{2}}^{\text{U14}}$ to check whether 0.4 mM Gdm^+^ is sufficient to obtain the maximum amount of kissing hairpin dimer. Both measurements were done at 80 MHz offset and show no difference in modulation depth. Thus, increasing the Gdm^+^ beyond 0.4 mM does not induce formation of more kissing hairpin dimers.


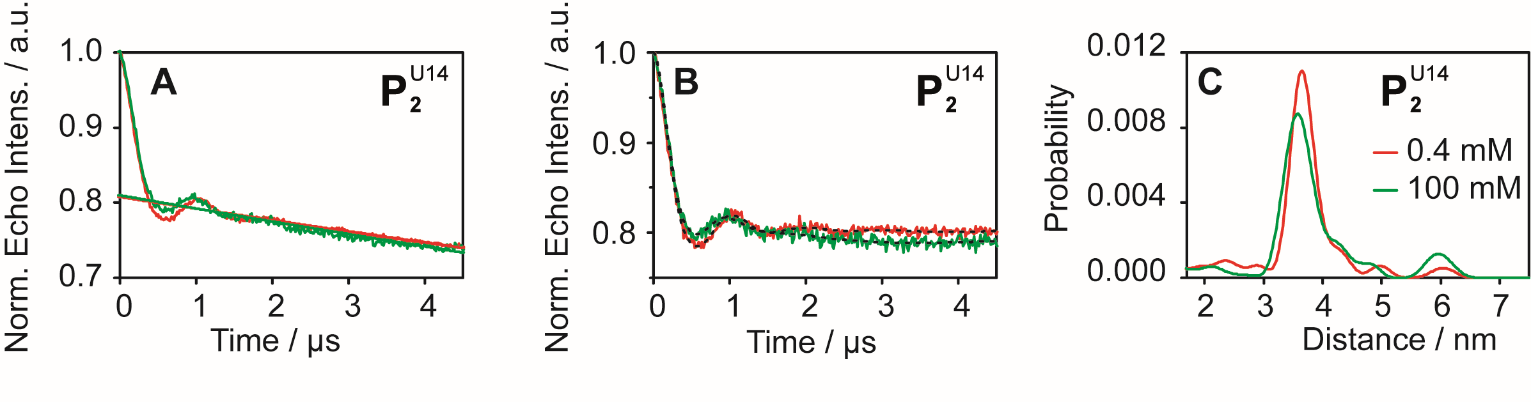


**Figure S14.** Data Analysis with DeerAnalysis of the 80 MHz offset of $\text{P}_{\text{2}}^{\text{U14}}$ in presence of 0.4 mM Gdm^+^ (red) and 100 mM Gdm^+^ (green). (A) Uncorrected PELDOR time traces. (B) Background corrected PELDOR time traces with background fit (black dashed line). (C) PELDOR-derived distance distribution.

5.3 Statistics of the Distance Distribution of $\text{P}_{\text{1}}^{\text{U20}}\mathbf{|}\text{P}_{\text{2}}^{\text{U14}}$ in Presence of Gdm^+^

**Table S8.** Analysis based on the peak amplitudes.

| Centre of the peak / nm | Height of the amplitude | Statistic |
| --- | --- | --- |
| 3.6 | 0.00346 | 1.3 |
| 4.4 | 0.00554 | 2.0 |
| 5.2 | 0.00371 | 1.3 |

**Table S9.** Analysis based on the peak area and Gaussian fits.

| Centre of the peak / nm | Area of the Gaussian Fit | Statistic |
| --- | --- | --- |
| 3.6 | 0.00183 | 1.0 |
| 4.4 | 0.00352 | 2.0 |
| 5.2 | 0.00776 | 1.3 |

**
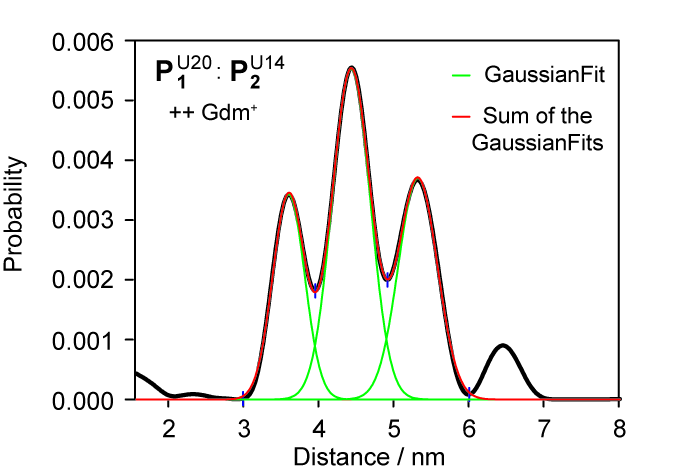
**

**Figure S15**. Gaussian Fit of the peaks of $\text{P}_{\text{1}}^{\text{U20}} | \text{P}_{\text{2}}^{\text{U14}}$ in presence of Gdm^+^.
